# Completion and determinants of a continuum of care in maternal health services in Benishangul Gumuz region: A prospective follow-up study

**DOI:** 10.3389/fpubh.2022.1014304

**Published:** 2022-11-04

**Authors:** Muluwas Amentie Zelka, Alemayehu Worku Yalew, Gurmesa Tura Debelew

**Affiliations:** ^1^Department of Public Health, College of Health Sciences, Assosa University, Assosa, Ethiopia; ^2^Department of Reproductive Health, School of Public Health, College of Health Sciences, Addis Ababa University, Addis Ababa, Ethiopia; ^3^Department of Biostatistics and Epidemiology, School of Public Health, College of Health Sciences, Addis Ababa University, Addis Ababa, Ethiopia; ^4^Department of Population and Family Health, Institute of Health, Jimma University, Jimma, Ethiopia

**Keywords:** Benishangul Gumuz region, maternal health, continuum of care, completion rate, Assosa

## Abstract

**Introduction:**

The provision of a continuum of care to women throughout pregnancy, labor, and after delivery has become a fundamental strategy for improving maternal and neonatal health. A better understanding of where the gaps are in seeking care along the pathway and what factors contribute to the gaps is required for successful program implementation. Hence, this study was targeted to determine the status and determinant factors of the completion rate of a continuum of care in maternal health services.

**Methods:**

A prospective follow-up study was conducted among 2,198 sampled pregnant women and followed for 11 months in Benishangul-Gumuz region. A multistage clustered sampling technique was employed to select the study participants. Data were collected *via* face-to-face interviews using a pretested, semi-structured questionnaire, and logbook registry. Collected data were edited, cleaned, and analyzed using *STATA software*. The multilevel regression model was used to examine the effects of individual- and community-level factors and expressed as AOR with 95% CI.

**Results:**

The completion rate of a continuum of care *via* visit-based, content-based, and space dimensions was 33.1, 20, and 37.2%, respectively. The enabling factors were having information on maternal health services (AOR = 2.25; 95% CI: 1.11–4.55), iron and folic acid supplementation (AOR = 2.58; 95% CI: 1.37–4.86), tetanus toxoid vaccination during pregnancy (AOR = 2.21; 95% CI: 1.39–3.52), having pregnant-related problems (AOR = 2.1; 95% CI: 1.15–3.71), dry and stimulate newborn (AOR = 2.61; 95% CI: 1.42–4.77), appropriate cord care (AOR = 2.01; 95% CI: 1.07–3.79), and immunizing newborn (AOR = 6.9; 95% CI: 3.79–12.59), whereas risk factors were ever having a stillbirth (AOR = 0.52; 95% CI: 0.32–0.85) and delay of 1st ANC initiation at 4–6 months of pregnancy (AOR = 0.45; 95% CI: 0.3–0.68).

**Conclusion:**

The completion rates *via* visit-based, content-based, and space dimensions were low. Different determinant factors which have a programmatically important implication were identified. Thus, interventions should focus on increasing women's awareness and improving the availability and accessibility of the whole packages of maternal health services to facilitate the completion rate.

## Introduction

Safe Motherhood Initiative (SMI) was launched in 1987 that encompasses key components of maternal health services, which have a significant role in the reduction of maternal and neonatal mortality and morbidity (1, 2). Maternal and newborn mortality are important indicators of the health status of a nation and socioeconomic situation. This is because, they are directly associated with a variety of factors such as maternal health services, quality and access to or availability of medical care services, socioeconomic conditions, and public health practices (2, 3). Hence, the continuum of care approach for maternal health is being championed as a means to ensure women receive essential services during pregnancy, delivery, and the postpartum period. The evidence reveals that the completion rate of a continuum of care in maternal health services in South Asia 25%; Sub-Saharan Africa 14% (4); Cambodia 60% (5); Ratanakiri Cambodia 5% (6); Northern Ghana 8% (7–9); Ghana 10.3% (10); Pakistan 27% (11); Tanzania 10% (12); Nepal 45.7% (13); Kenya 46.8% (14); and Rural Khammouane 6.8% (15) were low. As a result of the low completion rate of a continuum of care in maternal health services, every year about 15,000 mothers and 83,000 babies die in the countries, particularly in low-income countries including Ethiopia.

Recently, the concept of a continuum of care has been a core principle in maternal, newborn, and child health initiatives to save lives. However, access to care during and after delivery remains a challenging issue in the continuum of care framework (16). Continuity of care requires access to the service providers: at the family and community level, outpatient and outreach services, and clinical services throughout the lifecycle. The services provide at each time and place contribute to its effectiveness along with all the linked packages of maternal health services (17). Some of the evidence currently published in Ethiopia suggests that the completion rates of a continuum of care in maternal health services are extremely low. However, the study design used to collect information is a cross-sectional and secondary data, which does not give an accurate estimate of the completion rate and determinant factors (community and individual-level factors) of a continuum of care. Most of the studies on the completion rate of a continuum of care in maternal health services are Ethiopia Demographic and Health Survey (EDHS) 9.1% (18); Arba Minch district, Southern Ethiopia 9.7% (19); Northwest Ethiopia 21.6% (20); West Gojjam 12.1% (21), and Debre Berhan town 37.2% (22). The individual-level factors that influence the completion of a continuum of care in maternal health services are women's educational status (14, 22), maternal age (5, 14), early initiation of ANC services (19, 21, 22), birth preparedness and complication readiness (19, 22), being an employee (19), receiving adequate content of maternal health services (21), and other socio-demographic factors (14).

Assuring a continuity of care in maternal health services and offering key intervention packages are key strategies for improving maternal and neonatal health conditions (5, 23). Despite, in Ethiopia, the maternal mortality ratio is 412 deaths per 100,000 live births, and the neonatal mortality rate is 29 deaths per 1,000 live births, which are the highest in the world. Similarly, in Benishangul-Gumuz region, the neonatal mortality rate is 35 deaths per 1,000 live births which is higher than the national neonatal mortality rate (2). The strategies of a continuum of care are carried out during the pregnancy period, childbirth time, and postnatal period (17, 24). Each phase under the completion of a continuum of care follows a pathway from pregnancy to delivery to postpartum adding a certain value to ensure better health outcomes for mothers and newborns. Even though the maternal and child health program efforts are undergoing, it is critical to know and determine how to make the interventions more effective using cross-sectional and facility-based study design. Hence, effective implementation of the program to improve a continuum of care in maternal health services depends on having a good understanding of where the gaps are in seeking care along with the pathway from ANC to PNC services and what factors aggravate the occurrence of gaps. Instead of looking at maternal health services individually, a study from a perspective of a continuum of care using a prospective follow-up study design should give a clear picture of the pattern and barriers that affect women's continuation in receiving care from pregnancy to childbirth and after delivery.

Practically, the majority of previous studies are focused on individual-level characteristics and single components of maternal health services separately, rather than an integrated approach or continuity of services utilization. This may undervalue the relevance of taking into account community-based issues for developing effective maternal health initiatives in the study area and across the country. While multilevel regression modeling is appropriate for controlling the nesting effect of cluster variability at different levels, previous studies have relied on an ordinary logistic regression model which may not accommodate cluster variation within and between the clusters. As a result, the predictors may be underestimated or overestimated. Therefore, by overcoming the limitation of previous studies, the current study aimed to determine the completion rate of a continuum of care in maternal health services and also examine the effect of individual-level (*level – 1*) factors and community-level (*level – 2*) factors on the completion of a continuum of care in maternal health services.

## Methods and materials

### Study design and setting

This community and health facility-linked prospective follow-up study design was conducted in Benishangul-Gumuz region (BGR) from March 2020 to January 2021. The region is one of the 11 regional states constituting the Federal Democratic Republic of Ethiopia. The capital city of the region is Assosa which is located at a distance of 670 KMs Northwestern of Addis Ababa, the capital city of Ethiopia. Administratively, the region is structured into three zones, three city administrations, 21 districts, one special district, and 475 kebeles/clusters (439 rural and 36 urban). In 2021, the total population in the region was 1,173,123 in which 282,722 were females in childbearing age (15–49 years) (25). The total fertility rate in the region is 4.4 (26). The region represents around 4.6% of the total land area of Ethiopia and most of the people in the region are sparsely populated (27). The region has seven hospitals (six functional and one under construction), 67 health centers (60 functional and seven under construction), and 426 health posts (424 functional and two under construction) (25).

### Source population and study participants

The source population was pregnant women in the community at the time of the baseline survey in the region. Randomly selected pregnant women were the study participants.

### Sample size and sampling technique

Even though this study aimed to look at the completion rate and determinants of a continuum of care in maternal health services, it was a part of larger research work that encompasses six research objectives. Consequently, the sample size was calculated for all objectives, and the largest sample size was considered for all objectives. Thus, the sample size was determined for the effects of the completion of a continuum of care in maternal health services on the adverse birth outcomes, which was considered for this research work (28). Then, the sample size was calculated using STATA/MP 13.0 software based on the assumption of two population proportion formulas. The outcome variable was the adverse birth outcomes (stillbirth, neonatal death, and any illness within the neonatal period), and the predictor variable was the status of the continuum of care in maternal health service. No literature in Ethiopia supports determining the sample size for this objective. In rural India like Ethiopia, most births take place at homes and also high-risk practices are common (29). Unfortunately, neonatal mortality in Uttar Pradesh, India, (29 deaths per 1,000 live birth) (29) is almost similar to Ethiopia's neonatal mortality (29 per 1,000 live birth) (18). So, evidence stated in Uttar Pradesh, India, is used for sample size determination.

Accordingly, the proportion of the adverse birth outcomes, “*neonatal death,”* among women who complete a continuum of care in maternal health services is 4.29% (*P*_1_ = *0.0429)*, and the proportion of the adverse birth outcomes, “*neonatal death,”* among women who discontinuous utilization of maternal health services is 8.43% (*P*_2_ = *0.0843*) (29). A 95% confidence level and 80% power were used to detect a 4.14% difference between exposed and non-exposed groups. Moreover, the ratio of exposure to non-exposure pregnant women (r) was equal to 1:1 for the population allocation ratio; pooled population proportion (P) $=\frac{P_{1}+P_{2}}{1+r}$ was calculated (*P* = 0.0636), considering a design effect of 2 and a non-response rate of 10%. Based on this formula and assumption, the final sample size was 2,402. Then, 2,402 pregnant women were followed to measure the effect of a continuum of care in maternal health services on the adverse birth outcomes. Therefore, 2,402 pregnant women were used as the final sample size for this study.

The sampling technique was a multistage clustered sampling technique to recruit pregnant women for this study. Initially, the study area was stratified into three zones and three town administrations with one special *woreda*. In the first stage, of these stratified areas, two zones and one town administration were selected using a simple random sampling technique. Then after, seven districts/*woredas* and two town districts/*woredas* were randomly selected from the two zones and one town administration, respectively, as the second stage. Subsequently, at the third stage, 51 *Kebeles*/clusters were randomly selected from the selected districts/*woredas*.

A 1-month baseline census was conducted to identify pregnant women using a pregnancy screening criterion to prepare a sampling frame. Then, all pregnant women who resided in the selected *kebeles*/clusters were included in the study and then followed for an average of 11 months. During the baseline house-to-house survey, the health facilities that serve the selected study participants were enumerated and listed within their catchment areas. As a result, 46 health facilities were listed as candidates for the health facilities survey. Thus, three hospitals, 12 health centers, and 31 health posts were included in a health facility-based survey.

### Data collection process

Data collection instruments were prepared in English. The research instrument was adapted from Ethiopia Demography and Health Survey (2), National Technical Guidance for Maternal and Perinatal Death Surveillance and Response (30), MCH Program Indicator Survey 2013 (31), Survey tools conducted in Jimma Zone, Southwest Ethiopia (32), Survey tools conducted in Rural South Ethiopia (33), and other relevant different literature. Hence, to ensure the quality of data, training, pretest, supervision, and use of local languages were made.

### Data management and analysis

Collected data were coded and entered into *Epi_Info version 7.2.2.6* to design skipping patterns and minimize logical errors. Then, it is exported into *STATA Software version 14* for cleaning, editing, and analysis. Descriptive statistics were calculated for all variables. Univariable analysis crude odds ratio and 95% confidence interval were employed to select candidate variables (at *p* < 0.25) for multivariable analysis. A maximum likelihood estimate of the independent effect on the outcome variable was determined at the level of significance (*p* < 0.05). The household wealth index was calculated and categorized by using principal component analysis (PCA).

Before running the full model, effect modification or interaction effect at *p* < 0.1 and multi-collinearity effect between independent variables using variance inflation factors (VIF > 10%) were assessed. All independent variables included had VIF < 10, and the coefficient of the interaction terms was *p* > 0.1. Hence, there was no interaction and multi-collinearity effect. Since the sampling procedure for this study was a multistage clustered sampling procedure, due to cluster variability, a multilevel logistic regression model was applied to detect determinant factors of the completion of a continuum of care in maternal health services. So, for this study, “*Kebeles/Ketenas*” were considered as clusters, and also residents (being urban or rural) and household wealth index were considered as a level 2 factor. Women's individual-level variables were socio-demographic characteristics, obstetric characteristics, and information on maternal health services, and newborn health services were taken as a level-−1 factor. Log-likelihood ratio (LR) test was used to confirm the goodness of fit of the multilevel regression model that was found to be statistically significant such as data fit the model.

### Measurement and operational definition of variables

*Continuity of care after delivery:* Almost for two parameters, ANC and skilled delivery care are offered for pregnant women continuously according to a minimum recommendation of the World Health Organization (WHO) and also continue to receive a minimum package of PNC (1st PNC, 2nd PNC, 3rd PNC, and 4th PNC services).

*Continuity of care during childbirth:* Pregnant women receive a minimum package of ANC services and gave her birth at the health facility or attended by a skilled provider.

*Continuity of care during pregnancy:* A minimum package of the World Health Organization (WHO) offered for pregnant women by a skilled provider which encompasses a composite measure of four variables (1st ANC, 2nd ANC, 3rd ANC, and 4th ANC).

*Continuum of care in maternal health service:* A continuous utilization of maternal health services throughout their pregnancy time up to postnatal period: measured by a package of interventions consisting of a composite index of nine variables (1st ANC, 2nd ANC, 3rd ANC, 4^th^ ANC, Skill delivery care, 1st PNC, 2nd PNC, 3rd PNC, and 4th PNC services). Those women who received a full package of interventions are considered as “*completing continuum of care*” otherwise considered as “*discontinuation of care*.”

*Continuum of care in maternal health via space dimension* refers to an integration of maternal health services at the household, community, and facility level as well as referred to advanced level of care when needed.

*Continuum of care in maternal health via time dimension* refers to a situation where a woman and her newborn receive maternal health services along the continuum of care during pregnancy, childbirth, and postpartum.

*Discontinuation of maternal health services:* Missing at least one or more packages of intervention/s among composite measures of nine variables (1st ANC, 2ndANC, 3rd ANC, 4th ANC, Skill delivery care, 1st PNC, 2nd PNC, 3rd PNC, and 4th PNC services).

*Household wealth index:* Calculated by using household assets and income where the scoring factor of each asset is used to generate a wealth index through PCA.

## Results

### Response rate

A total of 2,439 pregnant women were enrolled from the selected 51 *kebeles*/cluster areas. Due to different reasons, around 241 pregnant women were lost from the study. After excluding loss-to-follow-up and incomplete data, 2,198 pregnant women were followed for 42 days after delivery and included in the current analysis.

### Health information, household wealth index, and accessibility of health facilities

The majority (90.9%) of study participants had information on maternal health services. The major sources of information were health workers 1,422 (71.2%), television 571 (28.6%), and radio 510 (25.5%). Regarding the household wealth index, 884 (24.5%) belonged to the 1st quintile (poorest) family members. Accessibility of health facilities for the study participants 1,888 (99.5%), 1,650 (75.1%), and 647 (29.4%) was found at a distance that took < 2 h to reach the health post, health center, and hospital, respectively (Table 1).

**Table 1:** Health information, household wealth index, and accessibility of health facilities in Benishangul-Gumuz region, Northwestern Ethiopia, March 2020–January 2021.

**Variables**	**Frequency**	**Percent (%)**
**Information on maternal health services**	
• Yes	1,997	90.9
**Source of information (** ***n** **=** **1,997, multiple response*** **)**	
• Health works TV Radio Community elder, leader and religion Others	1,422 571 510 124 4	71.2 28.6 25.5 6.2 0.2
**Household wealth index**	
• 1^st^ Quintile (Poorest) 2^nd^ Quintile (Poorer) 3^rd^ Quintile (Middle) 4^th^ Quintile (Richer) 5^th^ Quintile (Richest)	446 434 439 440 439	20.3 19.7 20.0 20.0 20.0
**Time to reach health post** ***via*** **foot** **(*****n** **=** **1,898*****)**	
< 1 Hours• ≥1 Hours	1,888 10	99.5 0.5
**Time to reach health center** ***via*** **foot** **(*****n** **=** **1,898*****)**	
< 2 Hours• ≥2 Hours	1,650 548	75.1 24.9
**Time to reach hospital** ***via*** **foot** **(*****n** **=** **1,898*****)**	
• < 2 Hours 2–6 Hours >6 Hours	647 974 577	29.4 44.3 26.3

### Past obstetric characteristics

The past obstetric history of women was assessed by the women's responses. Accordingly, the study revealed that 874 (35.2%) were married at an early age (below 18 years old) with a mean (±SD) of 18.13 ± 2.42, and 797 (36.3%) were first pregnant at teenage age (below 19 years old) with a mean (±SD) of 19.59 ± 2.62. Less than half, 882 (40.1%) had greater or equal to four gravidities; 610 (37.6%) ever had live births between 2 and 3 live births; 178 (10.78%) ever possessed a history of stillbirth, and 212 (12.8%) ever experienced a history of abortion. Almost two-thirds, 1,125 (68.1%) were given birth at the health facility; of them, 1,542 (93.4%) were given birth *via* spontaneous vaginal delivery. Based on the women's responses, 321 (19.4%) were having a history of pregnant-related problems during pregnancy. Among them, vaginal bleeding 104 (32.4%), severe headache 179 (55.8%), severe abdominal pain 139 (43.3%), and drowsiness 186 (57.9%) were common pregnant-related problems detected (Table 2).

**Table 2:** Past obstetric history of study subjects in Benishangul-Gumuz region, Northwestern Ethiopia, March 2020–January 2021.

**Variables**	**Frequency**	**Percent**
**Age at first marriage(years)**	
• < 15 15–17 ≥18	120 654 1,424	5.5 29.8 64.8
**Age at first pregnancy (years)**	
• < 16 16–18 ≥19	76 721 1,401	3.5 32.8 63.7
**Gravidity (number of pregnancy)**	
• 1 2–3 ≥4	547 769 882	24.9 35.0 40.1
**Number of live birth ever had (** ***n** **=** **1,622*** **)**	
• 1 2–3 ≥4	463 610 549	28.5 37.6 33.8
**Number of stillbirth ever had (** ***n** **=** **178*** **)**	
• 1 ≥2	135 43	75.8 24.2
**Ever had abortion**	
• Yes	212	12.8
**Frequency of abortion ever had (** ***n** **=** **212*** **)**	
• 1 ≥2	184 28	86.8 13.2
**Place of delivery for previous pregnancy (** ***n** **=** **1,651*** **)**	
• Home Health post Health center Hospital	426 358 655 212	25.8 21.7 39.7 12.8
**Mode of delivery for previous delivery (** ***n** **=** **1,651*** **)**	
• Spontaneous vaginal delivery Instrumental assisted delivery Operative abdominal delivery (C/S) Destructed vaginal delivery	1,542 68 36 5	93.4 4.1 2.2 0.3
**Pregnancy related problems ever had for previous pregnancy (** ***n** **=** **1,651*** **)**	
• Yes	321	19.4
**Pregnancy related complication for previous pregnancy (** ***n** **=** **321, multiple responses*** **)**	
• Drowsiness/tiredness Severe headache Severe abdominal pain Vaginal bleeding Facial swelling Persistent vomiting Hand swelling Others	186 179 139 104 59 51 48 2	57.9 55.8 43.3 32.4 18.4 15.9 15.0 0.6

### Continuum of care in maternal health services

#### The pattern of key maternal health care and packages

ANC service utilization is an entry point of a continuum of care. Hence, patterns of ANC visits were 1st ANC 1,919 (87.3%), 2nd ANC 1,815 (82.6%), 3rd ANC 1,674 (76.2%), and 4th ANC 1,453 (66.1%). The key interventions received during the ANC contacts were informed the danger signs of pregnancy 1,740 (79.2%), blood pressure measured 1,701 (77.4%), and iron folic acid supplementation 1,677 (76.3%). Similarly, the prevalence of skilled delivery services was 58.3%. Finally, the frequency of PNC visits was 1,783 (86.3%), 1,545 (74.8%), 1,373 (66.5%), and 1,210 (58.6%) of women attended for 1st PNC, 2nd PNC, 3rd PNC, and 4th PNC services, respectively. The key services received during the PNC visits were immunization of babies 1,692 (81.9%), counseling on proper nutrition 1,516 (73.4%), and breastfeeding education 1,436 (69.5%) (Table 3).

**Table 3:** Pattern of key maternal health services, obstetric characteristic and newborn care of study participants in Benishangul-Gumuz region, Northwestern Ethiopia, March 2020–January 2021.

**Variables**	**Frequency**	**Percent**
**Visit of ANC received during last pregnancy**	
• 1^st^ ANC contact 2^nd^ ANC contact 3^rd^ ANC contact 4^th^ ANC contact	1,919 1,815 1,674 1,453	87.3 82.6 76.2 66.1
**Key interventions received during ANC contact (** ***n** **=** **1,919, multiple response*** **)**	
• Informed on danger sign of pregnant Blood pressure measured Iron foliate supplementation Nutritional counseling Urine sample taken Blood sample taken Protection of birth from tetanus Other	1,740 1,701 1,677 1,623 1,607 1,578 1,562 22	79.2 77.4 76.3 73.8 73.1 71.8 71.1 1.0
**Delivery services for last delivery**	
• Skilled care Unskilled care	1,281 917	58.3 41.7
**Duration of labor**	
• < 12 B/n 12–24 h >24 h	1,457 458 150	70.6 22.2 7.3
**Pregnant related problems during labor**	
• Yes	295	14.3
**Gestational age at birth (*****n*** **=** **2,065)**	
• Preterm (< 37 weeks) Term (≥37 weeks)	240 1,825	11.6 88.4
**Time of premature rupture of membrane** **(*****n*** **=** **2,065)**	
• ≤ 1 h 1–12 h >12 h	814 1,132 94	39.9 55.5 4.6
**Component of PNC contact, she received** **(*****n** **=** **2,065*****)**	
• 1^st^ contact of PNC services 2^nd^ contact of PNC services 3^rd^ contact of PNC services 4^th^ contact of PNC services	1,783 1,545 1,373 1,210	86.3 74.8 66.5 58.6
**The key interventions offered during postnatal period (** ***n** **=** **2,065, multiple response*** **)**	
• Immunization of baby Counseling on proper nutrition Breast feeding education Physical examination Family planning services Other	1,692 1,516 1,436 1,248 1,074 30	81.9 73.4 69.5 60.4 52.0 1.5
**Newborn care during postnatal period** **(*****n** **=** **2,065, multiple response*****)**	
• Appropriate cord care Initiate breast feeding within 1 h Dry and stimulate newborn Inject vitamin k	1,666 1,644 1,593 1,258	80.7 79.6 77.1 60.9

#### Continuum of care via the time dimension

**Figure 1:**
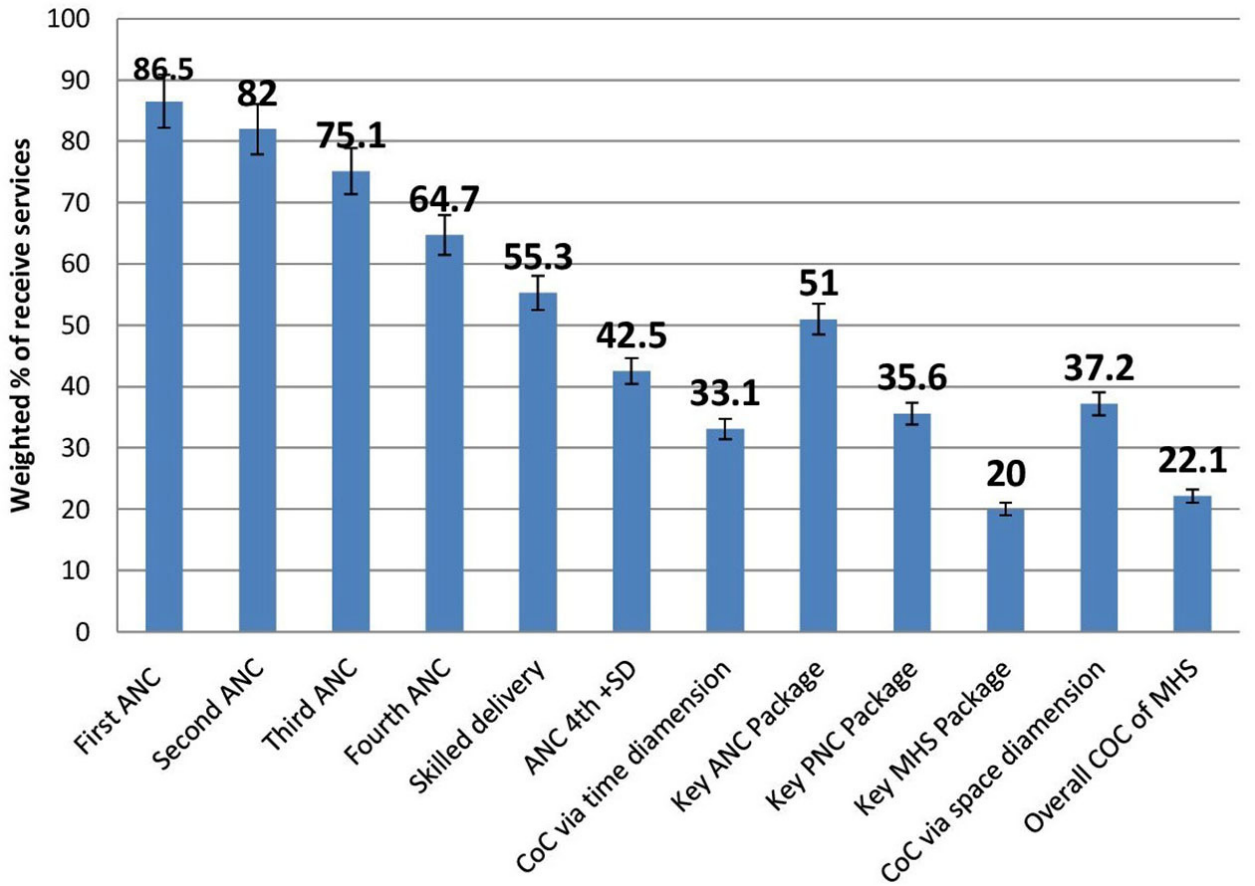
Level of continuum of care in maternal health services among the study subject in Benishangul Gumuz region, Northwestern Ethiopia, March 2020–January 2021.

After taking into account the cluster variation, 86.5% (95% CI: 86.2%, 86.8%) of women received the 1st ANC visit; however, it was only 64.7% (95% CI: 64.3%, 65.1%) continued for the 4th ANC visit. Of them, 42.5% (95% CI: 42.1%, 42.8%) continued and received both 4th ANC and skilled delivery care. Finally, only 33.1% of women continued with postnatal care services and completed the whole visits of the continuum of care in maternal health services *via* time dimension at 95% confidence interval (32.8–33.5%). Regarding continuity of key services, the completion of full packages of ANC services was 51.0% (95% CI: 50.7–51.4%), and the completion of full packages of PNC services was 35.6% (95% CI: 35.3–36.0%). Thus, the completion rate of all key services of maternal health services was 20.0% (95% CI: 19.7–20.4%). Therefore, the overall completion of a continuum of care in maternal health services *via* both time dimension and space dimension was 22.1 (95% CI: 21.8–22.5) (Figure 1).

#### Space dimension of the continuum of care

The space dimension is another parameter of the continuum of care in maternal health services; 1,706 (77.6%) of the respondents stated that maternal health services are offered at the household level. In the meantime, 1,935 (88.0%) of women linked to the health facility during home visits and the type of health facilities linked for maternal health services were health post 687 (35.5%), health center 1,121 (57.9%), and hospital 127 (6.6%). However, some of the women were not linked to the health facility because of different reasons: Women did not volunteer to go to the health facility 64 (24.3%), cultural forbidden 72 (27.4%), and the health workers did not tell her 92 (35.0%). More than half, 1,248 (56.8%) of the respondents mentioned that maternal health services are offered by health workers, trained birth attendants (TBA), and the health development army (HDA) at the community level. Moreover, 1,691 (76.9%) of the respondents described that maternal health services are integrating with other health promotion and disease prevention programs. The mechanisms used to increase the utilization and completion rate of a continuum of care in maternal health services were health workers linked pregnant women with community support 1,756 (79.9%), and declare community advocacy and sensitization 1,723 (78.4%). The overall completion rate of the continuum of care in maternal health services *via* space dimension was 37.2 % (95% CI: 35.2–39.3) (Table 4).

**Table 4:** Space dimension of continuum of care in maternal health services of the study subjects in Benishangul-Gumuz region, Northwestern Ethiopia, March 2020–January 2021.

**Variables**	**Frequency**	**Percent**
**Maternal health services offer at home level (*****n*** **=** **2,198)**	
• Yes	1,706	77.6
**Women linked to the health facility during home visiting (*****n*** **=** **2,198)**	
• Yes	1,935	88.0
**Type of health facility did the linkage made (*****n*** **=** **1,935)**	
• Health post/clinic Health center Hospital	687 1,121 127	35.5 57.9 6.6
**The main reasons for HWs didn't link to the health facility (*****n*** **=** **263)**	
• The health worker did not tell me any things Cultural forbidden to go to the health facility She didn't volunteer to go to the health facility She belief that at normal condition Other	92 72 64 44 10	35.0 27.4 24.3 16.7 3.8
**Maternal health services offered at the community level by health workers, TBA and TTBA (*****n*** **=** **2,198)**	
• Yes	1,248	56.8
**Maternal health service integrated with other health promotion and disease prevention services (*****n*** **=** **2,198)**	
• Yes	1,691	76.9
**Health work linked with community health support for maternal health services (*****n*** **=** **2,198)**	
• Yes	1,756	79.9
**Community advocacy and sensitization on maternal health services (*****n*** **=** **2,198)**	
• Yes	1,723	78.4
**Presence of community support to improve maternal health services within the community (*****n*** **=** **2,198)**	
• Yes	1,515	68.9
**Overall composite index category level of space dimension**	
Discontinuity of services *via* space *( ≤ 6 scores*)	1,380	62.8
Completion of services *via* space(*=7 scores*)	818	37.2

### Association between women's characteristics and completion of a continuum of care in maternal health services

Even though a total of 2,198 pregnant women were enrolled and interviewed within the study period, only 731 (33.3%) of women completed a continuum of care in maternal health services *via* time dimension. Of them, 518 (70.9%) resided in rural areas, 289 (39.5%) belonged to the age group between 25 and 29 years, 550 (75.2%) were Muslim religion followers, 718 (98.2%) were married, 593 (81.1%) were housewife in their occupation, 17 (2.3) were teenage marriage, 16 (2.2) were early pregnancy, and 97 (96.3%) had information on maternal health services. All basic women characteristic variables were had highly statistical significant association with completion of a continuum of care *via* time dimension at *p* < 0.01 (Table 5).

**Table 5:** Association between basic women characteristics vs. completion of continuum of care in maternal health services in Benishangul-Gumuz region, Northwestern Ethiopia, March 2020–January 2021.

**Variable**	**Completion of COC** ***via*** **time dimension**			**X^2^/Fisher test (*p*–Value)**
	**No [*n* (%)]**	**Yes [*n* (%)]**	**Total [*n* (%)]**	
**Place of residents**	
• Urban Rural	582 (39.7) 885 (60.3)	• 213 (29.1) 518 (79.9)	795 (36.2) 1,403 (63.8)	• *X^2^ = 23.453* *p < 0.0001*
**Age (years)**	
• 15–19 20–24 25–29 30–34 35–39 40–45	147 (10.0) 392 (26.7) 506 (34.5) 278 (19.0) 120 (8.2) 24 (1.6)	39 (5.3) 216 (29.5) 289 (39.5) 122 (16.7) 57 (7.8) 8 (1.1)	186 (8.5) 608 (27.7) 795 (36.2) 400 (18.2) 177 (8.1) 32 (1.5)	*X^2^ = 19.938* *p = 0.001*
**Religion**	
• Muslim Orthodox Others	922 (62.6) 456 (75.4) 89 (73.6)	550 (75.2) 149 (20.4) 32 (4.4)	1,472 (67.0) 605 (27.5) 121 (5.5)	*X^2^ = 35.313* *p < 0.0001*
**Marital status**	
• Married Single Others	1,384 (94.3) 74 (5.0) 9 (0.6)	718 (98.2) 10 (1.4) 3 (0.4)	2,102 (95.6) 84 (3.8) 12 (0.6)	*X^2^ = 21.155* *p < 0.0001*
**Woman occupational status**	
Housewife Governmental employee Merchant Student Others	1,140 (77.7) 115(7.8) 75 (5.1) 112(7.6) 25 (1.7)	593 (81.1) 70 (9.6) 19 (2.6) 27 (3.7) 22 (3.0)	1,733 (78.9) 185 (8.4) 94 (4.3) 139 (6.3) 47 (2.1)	*X^2^ = 25.556* *p < 0.0001*
**Age (year) at first marriage**	
• 10–14 15–17 ≥18	103 (7.0) 453 (30.9) 911 (62.1)	17 (2.3) 201 (27.5) 513 (70.2)	120 (5.5) 654 (29.8) 1,424 (64.8)	*X^2^ = 26.494* *p < 0.0001*
**Age (year) at first pregnancy**	
• 10–14 15–17 ≥18	60 (4.1) 506 (34.5) 901 (61.4)	16 (2.2) 215 (29.4) 500 (68.4)	76 (3.5) 721 (32.8) 1,401 (63.7)	*X^2^ = 12.670* *p = 0.002*
**Information on maternal health**	
• No Yes	174 (11.9) 1,293 (88.1)	27 (3.7) 704 (96.3)	201 (9.1) 1,997 (90.9)	*X^2^ = 39.171* *p < 0.0001*

### Determinant factors affecting the completion of a continuum of care in maternal health services

The determinant factors affecting the completion of the continuum of care were identified by using a multilevel logistic regression model. Before running the full model, ICC (ρ) was calculated in the empty model for the outcome to decide whether the data fit a multilevel model or not. Then after, ICC/*rho* (ρ) was calculated as a full model for the outcome to detect the variability attributed to clusters after controlling the individual level.

Rho (ρ)/ICC was calculated for the completion rate of a continuum of care in the empty model, and it was found to be 0.32 indicating that 32% of the variation was contributed by cluster variations. The test of preference of log-likelihood *vs*. logistic regression was also statistically significant (*P* < 0.0001). Then, the full model was run by including both the cluster-level and individual-level variables and the ICC (ρ) was reduced to 0.28. This again indicated that 28% of the variation was attributed to cluster-level variables. The preferred log-likelihood *vs*. logistic regression was statistically significant (*P* < 0.0001). Hence, this is suggesting that the preferred model for this outcome variable was multilevel logistic regression (Table 6).

**Table 6:** Parameter of odd ratio and test of goodness of fit for mixed-effect multilevel models, Benishangul-Gumuz region, Northwest Ethiopia, 2021.

**Models**	**Fixed intercept –cons (95%CI)**	**Random effect as Level-2 variance var [-cons (95%CI)]**	**Intra-class correlation coefficient: ICC(ρ)**	**Log likelihood (LR)-deviance**	**Significance of LR test vs. Logistic regression (*P*-value)**
**Continuum of care**					
• Empty model • Full model^a^	• 0.39 (0.27, 0.56) • 0.001 (0.001,0.005)	• 1.57 (0.95, 2.59) • 1.35 (0.7, 2.6)	• 0.32 = 32% • 0.28 = 28%	• −1,243.9 • −699.8	• *P* < 0.0001 • *P* < 0.00001

P < 0.05 is statistically significant, and the data fit for the multilevel model.

a Included variables in the full model are resident, household wealth index, age of women, educational status of women, occupation of partners, information on MHS, age at first pregnancy, ever had stillbirth, availability of MHS, time of 1^st^ ANC visit, ANC providers, IFA supplementation and TT vaccination during pregnancy, duration of labor, pregnant related problems, immediate newborn care practices, GA at birth and time of premature rupture of membrane.

After adjusting for confounders in the final two-level mixed-effect model, among the cluster-level variables, place of residence and household wealth index were not statistically significant associations with the completion of a continuum of care. However, among the individual-level variables, different factors which had programmatically important showed statistically significant association with the completion of a continuum of care in maternal health services.

The odds of completing the continuum of care in maternal health services among women who had any information on maternal health services (AOR = 2.25; 95% CI: 1.11, 4.55) were two times higher than among women who did not have information on maternal health services. In contrarily, the odds of completing the continuum of care among women who had a history of stillbirth (AOR = 0.52; 95% CI: 0.32, 0.85), women who delayed initiating the first ANC visit, 4–6 months of gestational age (AOR = 0.45; 95% CI: 0.3, 0.68), and after 6 months of gestational age (AOR = 0.15; 95% CI: 0.05, 0.43) were 48, 55, and 85%, respectively, lower than among women within their counterpart.

The odds of the completion rate of a continuum of care in maternal health services among women who attended ANC follow-up by skilled providers (AOR = 1.37; 95% CI: 1.02, 2.48) were 1.37 times higher than among women who did not attend ANC follow-up by skilled providers. Moreover, the odds of completing a continuum of care among women who received iron and folic acid supplementation during pregnancy (AOR = 2.58; 95% CI: 1.37, 4.86) were 2.58 times higher than among women who did not receive iron and folic acid supplementation during pregnancy. Similarly, the odds of completing a continuum of care in maternal health services among women who were vaccinated with a tetanus toxoid (TT) vaccine during pregnancy (AOR = 2.21; 95% CI: 1.39, 3.52) were 2.21 times higher than among women who did not receive tetanus toxoid (TT) vaccination during pregnancy.

In addition, the odds of completing the continuum of care in maternal health services among women whose newborns were immunized with the vaccine within the postnatal period (AOR = 6.9; 95% CI: 3.79, 12.59) were 6.9 times higher than among women whose newborns did not vaccinate with the vaccine. Moreover, the odds of the completion rate of a continuum of care in maternal health services among women whose newborns were dry and stimulate immediately after delivery (AOR = 2.61; 95% CI: 1.42, 4.77) and properly caring umbilical cord (AOR = 2.01; 95% CI: 1.07, 3.79) were two times higher than among women within their counterpart. Finally, the odds of completing a continuum of care in maternal health services among women who had a pregnant-related problem during labor (AOR = 2.1; 95% CI: 1.15, 3.71) were two times higher than among women who did not have any pregnant-related problems during labor (Table 7).

**Table 7:** Multilevel model analysis of determinants associated with the completion of continuum of care in maternal health services *via* time dimension, Benishangul-Gumuz region, Northwest Ethiopia 2021.

**Factors**	**Level of continuum of care**		**aOR 95%CI**
	**Discontinuous**	**Completion**	
**Level−2 (Community level) variables**	
**Place of residents**	
Urban Rural	582 (73.21) 885 (63.08)	213 (26.79) 518 (36.92)	1 1.69 (0.81, 3.55)
**Household wealth index**	
1^st^ Quintile (poor) 2^nd^ Quintile (middle) 3^rd^ Quintile (rich)	486 (66.3) 465 (63.35) 516 (70.59)	247 (33.7) 269 (36.65 215 (29.41)	1 1.09 (0.74, 1.61) 1.1 (0.61, 1.98)
**Leve-1 (individual level) variables**	
**Age (years)**	
< 20 20–29 ≥30	147 (79.03) 898 (64.01) 422 (69.92)	39 (20.97) 505 (35.99) 187 (30.71)	1 2.86 (0.87, 9.37) 1.99 (0.6, 6.65)
**Woman education level**	
No formal education Primary school High school Tertiary education	890 (66.67) 285 (67.54) 172 (69.35) 120 (62.18)	445 (33.33) 137 (32.46) 76 (30.65) 73 (37.82)	1 0.99 (0.59, 1.49) 1.07 (0.59, 1.92) 1.48 (0.79, 2.76)
**Partner occupational status**	
Governmental employee Others	266 (63.48) 1,118 (66.43)	153 (36.52) 565 (33.57)	1 0.99 (0.66, 1.6)
**Information on MHS**	
Yes	1,293 (64.75)	704 (35.25)	**2.25 (1.11, 4.55)**
**Age at first pregnancy**	
< 19 ≥19	566 (71.02) 901 (64.31)	231 (28.98) 500 (35.69)	1 1.18 (0.86, 1.60)
**Stillbirth ever had**	
Yes	130 (73.03)	48 (26.97)	**0.52 (0.32, 0.85)**
**Availability of MHS**	
Yes	1,365 (66.04)	702 (33.96)	1.33 (0.59, 3.0)
**Time of first ANC initiation**	
1–3 months of GA 4–6 months of GA After 6 months of GA	290 (50.88) 899 (67.59) 112 (84.85)	280 (49.12) 431 (32.41) 20 (15.15)	**1** **0.45 (0.3, 0.68)** **0.15 (0.05, 0.43)**
**Attendant of ANC services for current pregnancy**	
Skilled provider	1,118 (62.88)	660 (37.12)	**1.37 (1.02, 2.28)**
**Iron-folic acid supplementation**	
Yes	976 (58.20)	701 (41.80)	**2.58 (1.37, 4.86)**
**TT vaccinate during pregnancy**	
Yes	908 (58.13)	654 (41.87)	**2.21 (1.39, 3.52**)
**Duration of labor**	
< 12 B/n 12–24 h >24 h	954 (65.48) 295 (64.41) 85 (56.67)	503 (34.52) 163 (35.59) 65 (43.33)	1 1.41 (0.95, 2.08) 1.27 (0.6, 2.70)
**Pregnant related problem during labor for last delivery**	
Yes	181 (61.36)	114 (38.64)	**2.1 (1.15, 3.71)**
**Dry and stimulate newborn**	
Yes	907 (56.94)	686 (43.06)	**2.61 (1.42, 4.77)**
**Appropriate cord care practice**	
Yes	980 (58.82)	686 (41.18)	**2.01 (1.07, 3.79)**
**Initiate BF within 1 h**	
Yes	970 (59.0)	674 (41.0)	1.29 (0.73, 2.26)
**Inject vitamin k**	
Yes	697 (55.41)	561 (44.59)	1.37 (0.89, 2.09)
**Immunizing the newborn**	
Yes	1,009 (58.83)	706 (41.17)	**6.9 (3.79, 12.59)**
**Gestational age at birth**	
Preterm (< 37 weeks) Term (≥37 weeks)	133 (55.42) 1,200 (65.79)	107 (44.58) 624 (34.21)	1 0.64 (0.39, 1.05)
**Time of premature rupture of membrane before labor**	
≤ 1 h 1–12 h >12 h	500 (61.43) 766 (67.67) 51 (54.26)	314 (38.57) 366 (32.33) 43 (45.74)	1 0.76 (0.55, 1.05) 1.25 (0.56, 2.77)

The bold value indicates a statistically significant association (*p* < 0.05).

## Discussion

The completion rate of a continuity of maternal health services indicators is the global monitoring and reporting parameters that are used to measure the survival of women and newborns (34). This study found that 86.5% of women received the 1st ANC visit; however, only 64.7% of women continued for the 4th ANC visit. Of them, 42.5% of women continued and received both 4th ANC visits and skilled care, which is higher than the study done in PDHS (11), South Asia and Sub-Saharan Africa (4), Northern Ghana (8), Ratanakiri Cambodia (6), Arba Minch (19), and Northwest Ethiopia (20), but it is found to be lower than prior studies outside Ethiopia (5, 7, 10) and Debre Berhan town (22).

In this study, the overall completion rate of a continuum of care *via* time dimension was 33.1%. This finding is consistent with a study in Pakistan 27% (11) but higher than the studies from Ghana 7.9% (10), Tanzania 10% (12), Rural Khammouane 6.8% (15), EDHS (2016) 9.1% (18); Arba Minch 9.7% (19); Northwest Ethiopia 21.6% (20), and West Gojjam 12.1% (21). This variation may be due to the prior studies being a cross-sectional study design which has a full of recall bias that leads to underestimate the completion rate. In addition, in this study, the continuum of care is described in the form of space dimension that we have included the services offered during home visiting and campaigns as the measurement indicators. However, this finding is lower than evidence in Debre Berhan town 37.2% (22), CDHS 60% (5), Kenya 46.8% (14), Voucher and free services interventions areas in Kenya 56.1% (14) and Ghana (7). The reasons for the discrepancy may be due to a variation in socio-demographic and economic factors, the availability and accessibility of health facilities, study time, and design. Despite the Ethiopian government and the World Health Organization (WHO) are implementing many efforts on maternal and child health program such as distributing ambulance services, deploying community health extension workers and health workers, expansion of health infrastructure, and empowering political leaders to involve in maternal health services, still the progress of the completion rate of a continuum of care in maternal health services in Benishangul-Gumuz region is extremely low.

The coverage of contact-based continuity of care and content-based continuity of care is used as proxy indicators of the quality of maternal health services (34). According to WHO envisions, every pregnant woman and newborn receives core packages of services throughout the pregnancy, childbirth, and the postnatal period. However, in this study, only about half of the pregnant women received full packages of ANC services and one-third of the women received full packages of PNC services. The overall completion rate of content-based continuity of services was 20.0% in the study area, which was extremely low compared with the completion rate of visit-based continuity of care. This finding is consistent with the study done in Arba Minch (19), whereas this finding is lower than evidence from Ghana (10) and Sub-Saharan Africa (34) but it is higher than the study from West Gojjam zone (21). This discrepancy is due to the variation of measurement indicators that are used for the study. Generally, the completion rate is extremely low across the countries and abroad global. This is because globally agreed-upon measures of antenatal care (ANC), skilled birth attendance (SBA), and postnatal care (PNC) only capture the level of contact/visit with the health system and pay little attention to indicators of the actual core packages of maternal health services received by mothers and their newborns (34). This implies that achieving a visit-based continuum of care did not necessarily translate into the service-based continuum of care and vice versa (10).

Although both community- and individual-level characteristics are significantly important for program implications, in this study, only individual-level factors are detected. Those factors are more easily manageable and treatable, but, the community-level factors have no statistically significant association with the completion rate. This finding is consistent with the study done in West Gojjam, Ethiopia (21), and Ghana (7). However, evidence in Kenya reveals that the completion of the continuum of care in maternal health services was higher among women who resided in urban areas and belonged to the highest household wealth index (14). These may be the presence of community health extension workers, who are implementing maternal health services at the grass root level and providing the health services for the community irrespective of their wealth status and residence in Ethiopia.

This study found that women who had any information on maternal health services were more likely to complete a continuum of care in maternal health services *via* the time dimension, which is consistent with studies in Debre Berhan town (22), Northwest Ethiopia (20), and PDHS (11). Moreover, in this study, women who attended ANC follow-up by skilled providers were more likely to complete a continuum of care which is consistent with the evidence in Tanzania (12). The reason may be women, who had an experience with ANC follow-up, have access to information on the advantages and importance of maternal health services during ANC visits, and skill differences among service providers have an impact on the level of client satisfaction and the completion rate of a continuum of care in maternal health services. The evidence supported that getting health education on maternal health services and being satisfied with the service delivery were more likely to complete the pathway of maternal health services (20). Similarly, women who received iron and folic acid supplementation, tetanus toxoid (TT) vaccination during pregnancy, and immunized their newborns immediately after delivery were more likely to complete the pathway from ANC to PNC services in this study. This finding is also supported by studies conducted in Ethiopia (20, 21).

In this study, we found that women having pregnant-related problems during labor were three times more likely to complete a continuum of care. This finding is consistent with studies done in West Gojjam (21) and Tanzania (12), but it is contradicting with a study done in Ghana (10). This may be due to a variation in knowledge level on pregnant-related problems and the impact of problems on maternal and neonatal health outcomes. Similarly, in this study, women who had a history of stillbirth were less likely to complete the pathway of continuity of care which is also supported by different studies (10, 21). This is because women who encountered with psychological distress may be discontinued maternal health services.

In this study, women who delayed initiating the 1st ANC visit were less likely to complete the continuity from ANC to facility delivery to PNC. This finding is consistent with studies conducted in Ethiopia and different parts of the world (14, 15, 19, 21). The reason may be many essential core interventions are offered during ANC visits when the first ANC visit should be started as early as possible in the first trimester, which enhances the satisfaction of clients and increases the chance of using a skilled attendant at birth and also continued to receive the whole postnatal essential core interventions after childbirth.

### Policy and program implication of this study

This study came up with evidence that the majority of pregnant women initiate their first ANC visit. However, few women are completing the continuum of care along the pathway. These imply that the completion rate of a continuum of care in maternal health services is low. The main reasons for discontinuing the services are lack of knowledge, inaccessibility of the health facilities and shortage of supplies, lack of transportation services, and lack of skilled health workers. This pointed out the importance of raising the knowledge of women on the benefits of a continuum of care in maternal health service to enable every pregnant woman to complete the whole packages and services of a continuum of care in maternal health service. Furthermore, making the services available and accessible to the community is pivotal for the improvement of maternal and child health programs. Moreover, measurements of maternal health services were only capturing contact rather than the content of each measurement parameter (13). The implication of merely focusing on increasing coverage of recommended contacts within the health program rather than emphasizing essential packages of maternal health services is insufficient to reduce maternal and neonatal mortality and morbidity. Hence, the programmer designers and policymakers should target both contact- and content-based continuum of care in maternal health services *via* time and space dimension.

This study also addresses more programmatically important evidence at the community-level and individual-level factors that had a big contribution to the completion of the continuum of care in maternal health services. However, the community-level factors such as residents, household wealth index, and accessibility of health facilities had no statistically significant association. However, this does not mean that they are programmatically non-significant. These pointed to the importance of addressing community-level factors to increase the completion rate of a continuum of care. Furthermore, among the individual-level factors, different factors have programmatically most important and show statistically significant association with the completion of a continuum of care. Hence, interventions need to target community-level factors to improve the completion of a continuum of care *via* time and space dimensions and also target the individual-level factors to improve maternal and neonatal survival.

Therefore, this study gives a better understanding for the program designers and policymakers on where the gaps are in seeking care along the pathway and the factors that contribute to the continuum of care in maternal health services. This evidence is vital for the successful implementation of maternal and child health program and for planning the health program in future.

### Strength of the study

This study employed a longitudinal study design, which helps to measure the cause-and-effect relationship. This study used a large sample size, which results in high power and precision for multilevel analysis. Moreover, this study applied advanced statistical models (multilevel regression model) to handle cluster effects and identify factors at various levels.

### Limitations of the study

Mothers who had abortions, stillbirths, and neonatal deaths were not comfortable to respond the research questions properly, which might compromise the finding. The health facility data were collected and recorded by health professionals, which might lead to social desirability bias and might compromise the result. Moreover, some medical terms were difficult to translate exactly to local languages, which might affect the respondent understanding.

## Conclusion

The completion rate of a continuum of care in maternal health services *via* time (33.3%) and space dimension (37.2%) was low. Even though once women come to their first ANC visit, they were dropout from the pathway of continuity of maternal health services. Also, the completion rate of the essential packages of maternal health services (20%) was low. This implies that those who visit a health facility do not receive the basic package of maternal health services. This study shows that information on maternal health services, previous health facility delivery, ANC visit attended by skilled providers, offering iron and folic acid supplementation during pregnancy, vaccinating pregnant women with TT vaccine and immunizing the newborn, and proper immediate newborn care practice have positive factors that enhance completion rate of a continuum of care in maternal health services. However, history of stillbirth and delay to start ANC visit were negative factors that obscure the completion of continuity of care in maternal health services.

Therefore, interventions should focus on increasing women's awareness, improving the availability of packages of services at health facility particularly essential maternal health intervention packages, and improving service delivery by considering women's preferences and needs to increase their satisfaction. Those interventions are essential to increase the completion of continuum of care in maternal healthcare services *via* time dimension, content-based, and space dimension.

## Data availability statement

All original contributions presented in the study are included in the article/supplementary material.

## Ethics statement

The studies involving human participants were reviewed and approved by the Research Review and Ethics Committee (REC) of Addis Ababa University School of Public Health with protocol number SPH/3089/011 and ethical approval was acquired from the Institutional Review Board (IRB) of the College of Health Science, Addis Ababa University with protocol number 048/19/SPH. By deleting any identities from the questionnaire, confidentiality was maintained. The patients/participants provided both their verbal and written informed consent to participate in this study.

## Author contributions

All authors contributed to the conception, study design, execution, acquisition of data, analysis, interpretation, prepared and commented on the manuscript, gave final approval of the version to be published, agreed to submit to the journal, and agreed to be accountable for all aspects of the work.

## Funding

The research was funded by Addis Ababa University in collaboration with Assosa University.

## Conflict of interest

The authors declare that the research was conducted in the absence of any commercial or financial relationships that could be construed as a potential conflict of interest.

## Publisher's note

All claims expressed in this article are solely those of the authors and do not necessarily represent those of their affiliated organizations, or those of the publisher, the editors and the reviewers. Any product that may be evaluated in this article, or claim that may be made by its manufacturer, is not guaranteed or endorsed by the publisher.

## Abbreviations

ANC: antenatal care
AOR: adjusted odd ratio
AVD: assisted vaginal delivery
BGRS: Benishangul-Gumuz regional state
CDHS: Cambodia Demographic Health Survey
CI: confidence interval
COC: continuum of care
CS: cesarean section
EDHS: Ethiopia Demographic and Health Survey
HAD: Health Development Army
HEWs: health extension workers
HF: health facility
HWs: health workers
ICC: intraclass correlation
ID: institutional delivery
IFA: iron and folic acid
IRB: institutional review board
LR: log-likelihood ratio
MCH: maternal and child health
MHS: maternal health service
MPDSR: Maternal and Perinatal Death Surveillance and Responses
OR: odd ratio
PCA: principal component analysis
PDHS: Pakistan Demographic and Health Survey
PNC: postnatal care
REC: Research Review and Ethics Committee
SD: skilled delivery
SMI: Safe Motherhood Initiative
SPSS: Statistical Package Software for Social Sciences
SVD: spontaneous vaginal delivery
TBA: traditional birth attendant
TT: tetanus toxoid
TTBA: Trained Traditional Birth Attendant
TV: television
VIF: variance inflation factor
WHO: World Health Organization.
